# Women’s suggestions on how to improve the quality of maternal and newborn care during the COVID-19 pandemic in Italy: A co-occurrence network analysis

**DOI:** 10.7189/jogh.13.06013

**Published:** 2023-05-05

**Authors:** Sara Geremia, Emanuelle Pessa Valente, Ilaria Mariani, Paolo Dalena, Marzia Lazzerini

**Affiliations:** WHO Collaborating Centre for Maternal and Child Health, Institute for Maternal and Child Health – IRCCS “Burlo Garofolo” – Trieste

## Abstract

**Background:**

Recent evidence revealed significant gaps in the quality of maternal and newborn care in the World Health Organization (WHO) European Region (EUR) countries. Collecting and analyzing women’s views on their needs and priorities is crucial for developing actions to improve the quality of maternal and newborn care. With this study from the IMAgiNE EURO Project, we aimed to add to previous quantitative studies by analysing emerging themes from women’s suggestions on how to improve the quality of maternal and newborn care during facility-based birth in Italy during the COVID-19 pandemic.

**Methods:**

We collected data from mothers giving birth during the coronavirus 2019 (COVID-19) pandemic using a validated online anonymous WHO standard-based questionnaire consisting of open-ended questions. Using a word co-occurrence network (WCON), we analysed responses in Italian from women who gave birth between March 2020 and March 2022. This approach entails a graphical representation of word pairings that frequently co-occur across sentences and compose clusters.

**Results:**

The texts, produced by 2010 women participating in the study, consisted of 79 204 words and 3833 sentences. Eight clusters emerged with WCON, the three largest of which were related to companionship during childbirth, breastfeeding support, and physical resources. The term “swab”, associated with other terms in the COVID-19 domain, had the highest degree of centrality, thus representing a core topic.

**Conclusions:**

The key emerging themes from women's suggestions can be used to shape policies to improve the quality of care for mothers and newborns. Our WCON analysis offers a valid approach to quickly screen large textual data on quality of care, providing a first set of major themes identified by clusters. As such, it could be used to improve documentation of service users’ suggestions promoting the engagement of both researchers and policymakers.

**Registration:**

ClinicalTrials.gov: NCT04847336.

Quality of maternal and newborn care lies at the heart of the World Health Organization (WHO) policy framework and strategy [1]. The coronavirus disease 2019 (COVID-19) pandemic has increased the burden on health systems and constrained the delivery of high-quality care [2]. Recent evidence highlighted major gaps in the quality of maternal and newborn care, even across the WHO European Region (EUR) [3-5]. Evidence from national surveys has shown a decrease in medical and social support for pregnant and breastfeeding women, but an increase in women's concerns about childbirth [6-8].

Improvement in the quality of maternal and newborn care requires taking action, including collecting and analysing women’s views on their needs and priorities [9,10]. The IMAgiNE EURO study aims to gather information on the hospital quality of maternal and newborn care from both the mothers' and hospital workers' perspectives during the COVID-19 pandemic, utilizing 80 WHO Quality Measures [9]. It involves the development, validation, and use in the WHO EUR of two questionnaires based on the WHO Standards for improving the quality of maternal and newborn care [3,4,9,11].

Documenting women’s suggestions around the time of childbirth during the COVID-19 pandemic is critical for both researchers and policymakers and can inform the implementation of women-centred health care. Women’s suggestions can easily be collected with surveys containing open-ended questions, but their thematic analysis is very time-demanding and becomes complicated when the amount of available texts increases, of which only a small sample gets analysed. Alternative methods are needed to analyse large data sets of users' feedback (e.g. mothers’ or patients’) more quickly, allowing researchers to extract key themes from large text data sets. Being able to analyse large text data sets may require more targeted quantitative or qualitative analysis (e.g. on specific themes) and help generate new evidence for improving the quality of maternal and newborn care. However, literature on this topic is very scarce. We aimed at using a text-mining technique to identify key emerging themes related to women’s comments on how to improve the quality of maternal and newborn care in Italy.

## METHODS

### Research design

We used the Standards for Reporting Qualitative Research (SRQR) to report this online survey-based study [12].

### Setting and population

We conducted this online survey in Italy between 18 November 2020 and 13 March 2022. Women who gave birth in an Italian facility from 1 March 2020 until the end of data collection on 13 March 2022 were eligible to participate. The exclusion criteria were: maternal death, perinatal death (including stillbirth), refusal to participate, having psychiatric or psychosocial problems leading to inability to fill in the questionnaire, belonging to an age group under 18 years, and language barriers.

### Data collection tools and methods

We used a validated anonymous questionnaire to collect data and recorded them using REDCap 8.5.21 (REDCAP, Vanderbilt University, USA; 2018) via a centralized platform, which has been described elsewhere [9]. Briefly, the questionnaire for mothers included 40 WHO Standards-based Quality Measures [5] guiding them through an analysis of their birth experience. Finally, the questionnaire included one open-ended question (“Do you have any suggestions to improve the quality of care provided at the facility where you gave birth?”) which was the main focus of our analysis.

We disseminated the survey following a setting-specific plan through social media, organizational websites, and local networks, which include mothers’ groups and non-governmental organizations (NGOs).

### Justification for data analysis methods

The collection of texts that emerged from the open-ended question consisted of 79 204 words and 3833 sentences. Conducting the analysis of said data is time- and resource-consuming when performed with classical qualitative research methods, such as thematic analysis [13]. Thus, we decided to use the word co-occurrence network (WCON) analysis (also known as semantic network analysis) [13,14] to analyse our large textual data set. WCON is a text mining approach that can be used to analyse textual data, allowing a graphical display of pairs of words frequently co-occurring across the text. It has been used previously to analyse large textual data from user-generated content, such as social media content and research data, including academic papers, interview transcripts, and open-ended survey responses [15-19]. WCON analyses the interactions between representative words and the structures that these interactions create, such as the strength of the links between words, the position of words within the network, and the patterns of densely connected clusters of words, enabling easy identification of themes through graphical representation.

### Sample size

Existing guidance and previous studies [13,20] do not allow for defining a precise sample size for WCON. According to Seveg et al. [13] “When the purpose of the study is to find the main themes and frames, a random sample of a few thousand units (be it sentences or posts) would often be sufficient”. There is no upper limit in the sample size, although larger samples allow for making general claims regarding a text’s thematic content.

### Data analysis: Pre-processing

Standard textual data pre-processing steps had to be followed before the WCON analysis to ensure that only representative words are included [21,22]. Therefore, we split the texts into single words (word tokenization) and sentences (sentence tokenization). The words were reduced to their unique dictionary key, called a lemma, and the part of speech related to each word was identified. We used the TreeTagger tool [23] to perform these steps, exploiting the lexicon and the collection of manually tagged training texts (with lemma and grammatical categories information) available for the Italian language.

Afterwards, we applied procedures for text cleaning. First, we used word tokenization to detect and exclude short and non-informative answers with less than four words from the analysis, such as “no”, “no suggestion”, or “nothing to add”. Next, we removed stop-words (i.e. extremely common and non-informative words such as “and”, “the”, and “to”), and numbers and symbols. The data were further cleaned using the grammatical category information to exclude indefinite adjectives (e.g. “none”, “some”), proper nouns, adverbs and some truncated or grammatically incorrect words (e.g. “min”, “ther’s”). By removing low-level information such as short answers and indefinite adjectives, we were able to focus on the most representative words to identify the key themes emerging from women's comments [22].

### Data analysis: Co-occurrence network analysis

Afterwards, we calculated the degree of word co-occurrence by Spearman’s rank correlation between all possible pairs of words across sentences [24]. As suggested by literature, we retained only word pairs that showed at least a 0.1 correlation coefficient [22] to limit the visualization of interactions between words with a very low degree of co-occurrence.

In a WCON visualization, the lines connecting each word represent the co-occurrence structure and each word is referred to by a point. To optimize data visualization, we graphically expressed the strength of the links between words through the opacity of the lines, with darker ones representing a higher degree of word co-occurrence.

Moreover, the WCON method identifies central words as those that appear linked with many others, thus conveying an important meaning in the text. For each word, the degree centrality is the number of lines connected to a word. Groups of points (i.e. words) with many internal lines and few external lines constitute word clusters. Words in the same cluster often share similar meanings and thus help researchers identify the semantic fields and prevailing themes within a text [25]. To optimize visualization, we displayed the size of each word point in proportion to its degree of centrality (i.e. words with more links were shown with bigger points, while their central positions in a word cluster indicated their centrality). We translated Italian terms in the WCON into English and displayed English labels for each point in the WCON graphical representation.

Four researchers with long-term experience in maternal and newborn health care and quality of care (SG, EPV, IM, ML) identified major themes that emerged from WCON analysis, labelling them with words reflecting their content. We ordered the themes by cluster size, defined as the sum of points in a cluster.

All analyses and the graphical representation of the network were performed with R (version 4.2.0, R Core Team, Vienna).

### Ethical approval

The survey was approved by the Institutional Review Board of the coordinating centre, the IRCCS “Burlo Garofolo” Trieste (IRB-BURLO 05/2020 15.07.2020), and by Instituto de Saúde Pública da Universidade do Porto (CE 20159), Norwegian Regional Committee for Medical Research Ethics (2020/213047), Bielefeld University ethics committee (2020–176), and Riga Stradins University Research Ethics Committee (22–2/140/2021 16.03.2021). Since this was an online survey that mothers joined voluntarily and no data elements that could disclose maternal identity were collected, formal approval was waived by the ethical committees from the other countries. Furthermore, we conducted this survey following the General Data Protection Regulation (GDPR). Before participating, we informed women of the study’s objectives and methods, and their right to opt-out. Each woman provided informed consent before responding to the questionnaire.

## RESULTS

### Characteristics of the analysed women

Out of 2143 women included in the analysis, 2010 were retained after the exclusion of cases providing short and non-informative comments. Characteristics of the sample are reported in **Table 1****.**

**Table 1:** Characteristics of the analysed women (n = 2010)

	Number of participants, n (%)
**Maternal age**	
18-24	40 (2.0)
25-30	407 (20.2)
31-35	877 (43.6)
36-39	496 (24.7)
≥40	190 (9.4)
**Year of birth**	
2020	1305 (64.9)
2021	631 (31.4)
2022	65 (3.23)
**Maternal educational level**	
None	1 (0)
Elementary school	69 (3.4)
High school	739 (36.8)
University degree or higher	1199 (59.6)
**Parity**	
1	1460 (72.6)
>1	550 (27.4)
**Mother giving birth in the same country where she was born**	
Yes	1916 (95.3)
No	94 (4.7)
**Mode of birth**	
Vaginal spontaneous	1245 (61.9)
Instrumental vaginal birth	169 (8.4)
Caesarean section	596 (29.7)
**Type of facility**	
Public	1880 (93.5)
Private	130 (6.5)
Type of health care providers who directly assisted the birth*	
Midwife	1733 (86.2)
Nurse	504 (25.1)
Student (i.e. before graduation)	103 (5.1)
Obstetrics registrar/medical resident (under post-graduate training)	314 (15.6)
Obstetrician-gynecologist doctor	1128 (56.1)
I don't know (health care providers did not introduce themselves)	213 (10.6)
**Other characteristics**	
Newborns admitted to the neonatal intensive care unit	184 (9.2)
Multiple births	25 (1.2)
Mothers admitted to the intensive care unit	5 (0.2)

*More than one possible answer.

Most of the women were highly educated (59.6% with a university degree or higher educational level) and aged between 25 and 39 years old (88.5%), with 877 (43.6%) being 31-35 years old. Ninety-four (4.7%) women did not give birth in their own country of birth. Most women (93.5%) gave birth in a public hospital; around one-third (27.4%) had a previous birth and more than half (56.1%) were assisted by an obstetrician-gynaecologist during delivery. Around two third of women (61.9%) had vaginal spontaneous birth; one-third had a caesarean birth (29.7%), 184 women (9.2%) reported that their child was admitted to the neonatal intensive care unit, and 25 (1.2%) had multiple births.

### Results of the WCON analysis

The texts consisted of 79 204 words and 3833 sentences, with a mean of 40 words and two sentences per woman.

**Figure 1:**
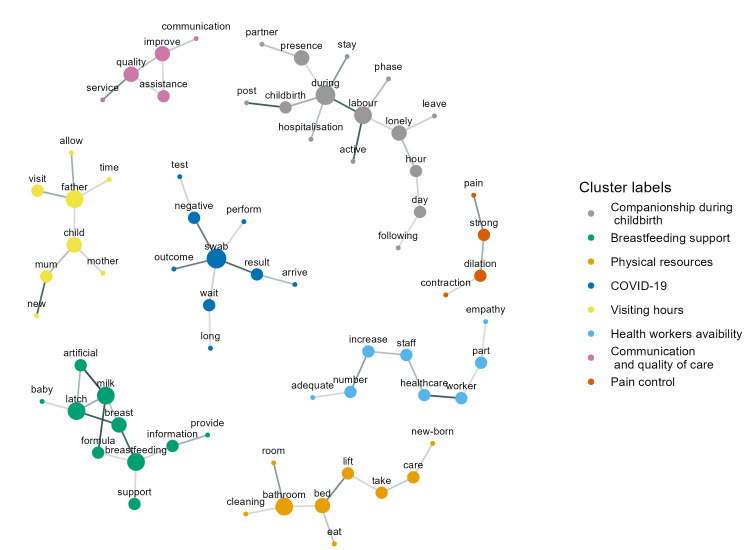
Women’s suggestions on how to improve the quality of maternal and newborn care: major themes in the co-occurrence network. The size of the points indicates node degree centrality; darker lines represent a higher degree of co-occurrence of pairs of terms. Cluster labels in the legend are in descending order of cluster size.

The resulting WCON graphic contained eight-word clusters, each detached from the other; it was presented as a network with all clusters shown without external links (**Figure 1**). The following eight major themes were identified by the clusters of words: companionship during childbirth, breastfeeding support, physical resources, COVID-19, visiting hours, health workers availability, communication and quality of care, and pain control. There was an average of 9.1 words per cluster, indicating a low variety of words used for the themes.

The term “swab”, included in the fourth cluster (COVID-19) showed the highest degree of centrality. The term “during” from the first cluster (companionship during childbirth) showed the same value of degree centrality but appeared in a less central position in its cluster. Other central words with a high degree of centrality included “father” (in the third cluster on visiting hours); “milk” and “breastfeeding” (in the second cluster on breastfeeding support).

## DISCUSSION

This report contributes to documenting the use of a text mining approach to analyse large textual data sets of user feedback. It made both content and methodological contribution to the existing literature by generating new evidence on women’s views on how to improve the quality of maternal and newborn care and by adopting a new approach for qualitative analysis.

All eight themes resulting from the analysis suggest actions that may improve care for mothers and newborns in Italy, thus being potentially useful for both policymakers and researchers. Policymakers should incorporate women's requests in their plans to improve health care, as expected for women-centred care [26].

Our findings appear aligned with existing qualitative research on the quality of maternal and newborn care which manually extracted information from texts [27-29], showing that what mattered most to women was support from a birth companion, together with the request for effective information and support from health professionals. Words in the physical resources cluster suggested that women give importance to the resources available in the facility where they give birth, which has also been reported by previous systematic reviews [27,28].

The high degree of centrality of the term “swab” in the COVID-19 cluster confirms the findings of previous studies which showed that COVID-19 influenced women's birth experience and perinatal mental health [6,7,30]. Notably, the data collection period saw changes in hospital care due to COVID-19 prevention measures. The centrality of the term “during” underscores the importance of the way time was conceptualized and reflects the findings of a previous study [31]. From women's birth stories, it emerged that the perception of time is a critical aspect of labour, with “perceived” time on average being longer than actual time in the experience of labour [31].

We can also conclude that our results align with the themes emerging from the “What women want” campaign recently conducted among 1.2 million women and girls from 114 countries [32], which include respectful, dignified and non-discriminatory care, increased, competent, and better-supported health workers, including midwives and nurses, and free or affordable medicines and supplies. Nevertheless, the aforementioned study insufficiently represented countries from the WHO EUR based on the response rate and country-specific analyses were not conducted. There are still very few studies from Italy on women's views on how to improve the quality of care [4,7,29,33], necessitating more studies in different settings. Quantitative data from the IMAgiNE EURO study on the maternal perspective have already been reported [3-5]. Our study complements existing ones by reporting on a qualitative analysis conducted on a large sample of mothers, thus adding new evidence. The IMAgiNE Euro Project is currently collecting data in more than 20 countries of the WHO European Region, and qualitative analyses of women's suggestion on how to improve the quality of care are expected from different settings, including Italy, Germany, Portugal, Norway and Switzerland, are expected soon.

Interestingly, “What women want” also investigated the opinions of midwives, enrolling 56 000 professionals in 101 countries and highlighting their demands for more and better-supported personnel, supplies and functional facilities, and the health of mothers and newborns. More studies should explore health professionals' perceptions on how to improve the quality of maternal and newborn health care. The IMAgiNE Euro Project includes a validated questionnaire for health workers [10], which so far enrolled about 7000 professionals. These data will be reported in an upcoming publication.

Limitations of the IMAgiNE EURO Study, e.g. selection bias, have been reported elsewhere [3]. As for any voluntary online survey, the sample may have been aligned toward women with a higher interest in participating and with internet access, but it is difficult to estimate how this selection may have affected study findings, particularly for our study. Notably, our study sample was large and the characteristics of the enrolled women were mostly aligned with those of women giving birth in Italy in the same period [34].

Although text mining cannot fully replace the traditional methods of qualitative analysis (i.e. with experts analyzing manually every single comment), this study suggests, in line with recent studies that have applied this methodology [14-16], that text mining may allow for quick screening of large data sets, thus providing an initial set of findings. WCON analysis is less time-consuming than manual coding, requires fewer resources, and can be considered a simple and straightforward approach that can be applied to any language, without the need to “teach” the computer the grammatical structure or the role of each word [14]. Additionally, findings from the WCON analysis contribute to identifying themes which may be further explored with other methods, such as thematic analysis or manual coding with traditional codebooks, allowing for a deeper understanding of context including hidden meanings [13]. These further analyses will be reported in later publications.

## CONCLUSIONS

This pilot study is valuable for health professionals, researchers, and policymakers, as it highlights key themes that emerged from women's suggestions on how to improve the quality of maternal and newborn care in Italy. It may also offer an example of an innovative approach that may facilitate the interpretation of findings from a large amount of available textual data, enabling quick comparison across different languages.
